# Lutein Attenuates Both Apoptosis and Autophagy upon Cobalt (II) Chloride-Induced Hypoxia in Rat Műller Cells

**DOI:** 10.1371/journal.pone.0167828

**Published:** 2016-12-09

**Authors:** Frederic K. C. Fung, Betty Y. K. Law, Amy C. Y. Lo

**Affiliations:** 1 Department of Ophthalmology, Li Ka Shing Faculty of Medicine, The University of Hong Kong, Hong Kong S.A.R; 2 State Key Laboratory of Quality Research in Chinese Medicine, Macau University of Science and Technology, Macau, China; 3 Research Centre of Heart, Brain, Hormone and Healthy Aging, Li Ka Shing Faculty of Medicine, The University of Hong Kong, Hong Kong S.A.R; Univerzitet u Beogradu, SERBIA

## Abstract

Retinal ischemia/reperfusion injury is a common feature of various retinal diseases such as glaucoma and diabetic retinopathy. Lutein, a potent anti-oxidant, is used to improve visual function in patients with age-related macular degeneration (AMD). Lutein attenuates apoptosis, oxidative stress and inflammation in animal models of acute retinal ischemia/hypoxia. Here, we further show that lutein improved Műller cell viability and enhanced cell survival upon hypoxia-induced cell death through regulation of intrinsic apoptotic pathway. Moreover, autophagy was activated upon treatment of cobalt (II) chloride, indicating that hypoxic injury not only triggered apoptosis but also autophagy in our *in vitro* model. Most importantly, we report for the first time that lutein treatment suppressed autophagosome formation after hypoxic insult and lutein administration could inhibit autophagic event after activation of autophagy by a pharmacological approach (rapamycin). Taken together, lutein may have a beneficial role in enhancing glial cell survival after hypoxic injury through regulating both apoptosis and autophagy.

## Introduction

Ocular diseases associated with retinal ischemia/reperfusion (I/R) injury lead to irreversible retinal cell death. In rodent models of retinal I/R injury induced by blockade of internal carotid artery, a remarkable cell loss was found in both ganglion cell layer (GCL) and inner nuclear layer (INL) in the retina [1]. Another study using an animal model of I/R injury induced by elevating the intraocular pressure also demonstrated an increase of apoptotic nuclei in INL [2].

Autophagy is an evolutionary conserved mechanism that allows the cell to degrade damaged proteins and intracellular organelles, maintaining cell homeostasis against nutrient deprivation and cellular stress [3]. Autophagy appears to be protective at the early onset of stress condition but can lead to cell death when excessively up-regulated. Produit-Zengaffinen *et al* and Piras *et al* reported that autophagy was triggered after I/R injury and resulted in further damage in retinal neurons [4,5].

Lutein is a member of xanthophyll family of carotenoids and it can be found in some dark leafy vegetables such as kale and spinach [6,7]. Lutein cannot be synthesized by the human body; therefore, it has to be obtained from the daily diet. Lutein consists of two hydroxyl groups, making it reacting more strongly with singlet oxygen than other carotenoids [8,9]. Lutein is also an efficient pigment for absorbing high energy blue light and protects photoreceptors from phototoxicity [10,11]; therefore lutein is known as a potent anti-oxidant and oxygen free radical scavenger. Clinically, lutein has been found to improve visual function and macular pigment optical density in patients with age-related macular degeneration (AMD) [12–14]. In addition, lutein has been shown to be neuroprotective in different retinal disease models including endotoxin-induced uveitis (EIU), light-induced retinal degeneration and retinal ischemia/reperfusion injury [1,15,16].

Műller cells are the principle glia of retina and they protect retinal neurons from excitotoxic damage as well as reactive oxygen species (ROS) induced by ischemia [17]. Műller cell gliosis responding to I/R injury results in retinal cell death [18]. We have previously shown that lutein administration protects retinal neurons from I/R injury *in vivo* and from oxidative stress *in vitro* [1,19]. *In vitro* hypoxia can be achieved by chemical-induced hypoxia or by oxygen-glucose deprivation (OGD) [20]. Cobalt (II) chloride (CoCl_2_), a common reagent to mimic the hypoxic/ischemic condition, induces the generation of reactive oxygen species (ROS) and in turn increases oxidative stress, resulting in cell death. It has been reported that ROS was induced in retinal ischemia and eventually led to retinal cell death [17]. We previously used CoCl_2_ to induce chemical hypoxia and demonstrated that lutein treatment attenuated the release of pro-inflammatory cytokines in a cultured rat Műller cell line (rMC-1) [21]. In the present study, we aim to further evaluate the anti-apoptotic effects of lutein in rMC-1 cells against CoCl_2_-induced hypoxic injury. In addition, as autophagy and apoptosis have been shown to be co-activated upon CoCl_2_ insult [22], we hypothesize lutein exerts a protective role in hypoxia-induced autophagy in rMC-1 cells.

## Materials and Methods

### Reagents

Lutein, cobalt (II) chloride, ammonium chloride, 3-Methyladenine (3-MA), and dimethyl sulfoxide (DMSO) were purchased from Sigma-Aldrich (St. Louis, MO). Rapamycin and Chloroquine were purchased from Enzo Life sciences. Lutein was dissolved in 100% DMSO and a stock solution (10mg/ml) was prepared and kept at -80°C until use. Lutein stock solution was further diluted in 0.01% DMSO as the working solution. Cobalt (II) chloride (10mM), ammonium chloride (1M), 3-MA (67mM), and chloroquine (60mM) were dissolved in water, respectively. Rapamycin was dissolved in DMSO at 500μM.

### Cell culture

An immortalized rat Műller cell (rMC-1) was routinely maintained in Dulbecco’s modified Eagle’s medium (Gibco, Carlsbad, CA) supplemented with 10% fetal bovine serum (FBS, Hyclone, Logan UT, USA), 100U/ml penicillin and 100ug/ml streptomycin (Gibco) [23]. Cells were grown in a humidified incubator of 95% air and 5% CO_2_ at 37°C and passaged when reached 80% confluent. Chemical-induced hypoxia was induced using cobalt (II) chloride (CoCl_2_) as described previously [21]. Briefly, rMC-1 cells were prepared in 6-well plates at a density of 2 x 10^5^ cells per well in DMEM and incubated 24 hours before treatment. Next, the cells were starved in DMEM with 1%FBS for 4 hours before inducing hypoxia. For dose dependent study, CoCl_2_ (300μM) was used to induce chemical hypoxia together with different doses of lutein (2.5, 5, 10 and 20 μM) or vehicle (0.01% DMSO) for 24 hours. For time dependent study, CoCl_2_ (300μM) was used to induce chemical hypoxia together with lutein (20 μM) or vehicle (0.01% DMSO) for designated time points. To examine the involvement of autophagy in CoCl_2_ -induced cell death, rMC-1 cells was treated with 3-MA (1mM) for 2 hours before CoCl_2_ treatment for 24 hours. To access the autophagic flux, rMC-1 cells were incubated with CoCl_2_ and ammonium chloride (NH_4_Cl) for 24 hours. To induce the mTOR-mediated autophagy, rMC-1 cells were exposed to rapamycin and chloroquine for 16 hours.

### Cell Viability Assay

Cell viability was examined using Celltiter 96 Aqueous Non-radioactive Cell Proliferation Assay (Promega, Madison, WT) according to the manufacturer’s protocol. Briefly, rMC-1 cells were seeded into 96-well plates as previously described [21]. Next, cells were treated as described above for designated time periods. After washes by 0.01M PBS, a mixture of 3-(4,5-dimethylthiazol-2yl)-5-(3-carboxymethoxyphenyl)-2-(4-sulfophenyl)-2H-tetrazolium (MTS) and phenazine methosulfate (PMS) was added to the wells and incubated for 3 hours at 37°C. Absorbance was measured with a microplate reader (ELX 800; Biotek Instruments, Winooski, VT) at 490nm. All results were obtained from five individual experiments in triplicates.

### Lactate dehydrogenase (LDH) cytotoxicity assay

CoCl_2_-induced cytotoxicity in rMC-1 cells leading to leakage of cytoplasmic enzyme lactate dehydrogenase (LDH) into culture medium was measured by Cytotoxicity Detection Kit (Roche) according to the manufacture’s instruction. CoCl_2_-induced hypoxia in rMC-1 cells was performed as described above. After treatments, both cell culture medium and cell lysate were incubated with the substrate containing Diaphorase/NAD^+^ as well as INT/sodium lactate for 15 minutes. Absorbance of LDH activities were measured at 490nm. Amount of LDH released from apoptotic cells was calculated as percentage of the total LDH activities. The results were obtained from five individual experiments with duplicate samples.

### Terminal Deoxynucleotidyl Transferase dUTP Nick End Labeling (TUNEL) assay

*In Situ* detection of apoptotic cells were performed using a DeadEnd^TM^ Fluorometric TUNEL System (Promega, Madison, WT). The assay was performed in accordance with the manufacturer’s protocol. Briefly, rMC-1 cells were grown on chamber slides and treatments were given as mentioned above. The cells were then fixed with 4% paraformaldehyde for 15 minutes at 4°C. After permeabilization with 0.1% Triton X-100 for 2 minutes, cells were incubated with the reaction mix containing terminal deoxynucleotidyl transferase (TdT) and nucleotides for 1 hour at 37°C. 4’,6-diamidino-2-phenlindole (DAPI) was used to stain the nuclei. Five nonoverlapping fields in each well were captured using a light microscope (Eclipse 80i Nikon, Tokyo, Japan) equipped with a digital camera (Diagnostic Instruments, Inc., Sterling Heights, MI). More than 250 cells in each field were counted using Image J software (National Institute of Mental Health, Bethesda, MD). Cells with TUNEL labeling co-localized with DAPI staining were counted as TUNEL-positive. The results were expressed as percentage of TUNEL-positive cells over the total number of cells counted from five individual experiments with duplicate samples.

### Western blot analysis

Whole cell lysates was prepared by addition of lysis buffer (50mM Tris PH 7.4, 150mM NaCl, 1% Triton X-100, 0.1% SDS, 1% Sodium deoxycholate). 10μg of protein lysate was separated by SDS-PAGE and then transferred to polyvinylidene difluoride membranes. After blocking with 5% non-fat milk, the membranes were incubated with primary antibodies, β-actin (1:5000; Chemicon, Temecula, CA), Bcl-2 (1:1000; Santa Cruz Biotechnology, Santa Cruz, CA), Bcl-X_L_ (1:1000; Cell Signaling Technology, Beverly, MA), Bax (1:1000; Cell Signaling Technology, Beverly, MA), Cleaved Caspase 3 (1:1000; Cell Signaling Technology, Beverly, MA), LC3 (1:1000; Cell Signaling Technology, Beverly, MA), P-p70S6K (1:1000; Cell Signaling Technology, Beverly, MA), p70S6K (1:1000; Cell Signaling Technology, Beverly, MA), p-AMPK (1:1000; Cell Signaling Technology, Beverly, MA), AMPK (1:1000; Cell Signaling Technology, Beverly, MA), p-mTOR (1:1000; Cell Signaling Technology, Beverly, MA), mTOR (1:1000; Cell Signaling Technology, Beverly, MA), p-ULK1(Ser757) (1:1000; Cell Signaling Technology, Beverly, MA) overnight at 4°C. After incubation with secondary antibody, signals were detected by ECL (Amersham Pharmacia Biotech, Arlington Height, IL) on a X-ray film, which was scanned and quantified using Image J software (National Institute of Mental Health Bethesda). Five independent experiments with duplicate samples were performed.

### Analysis of CoCl_2_-induced autophagy in rMC-1 cells

Assessment of autophagic activity in rMC-1 cells was performed using Cyto-ID^®^ Autophagy Detection kit (Enzo, Life Science). In brief, cells were grown on a chamber slide and treated with CoCl_2_ (300μM) as well as Lutein (20μM) for 24 hours. Cells co-treated with 0.5μM rapamycin and 120μM chloroquine for 16 hours were used as the positive control to induce autophagy and to identify the autophagic vesicles in rMC-1 cells. After washes by assay buffer, cells were incubated with Cyto-ID^®^ Green Detection Reagent for 45 minutes at 37°C. Hoechst 33342 was used as a nuclei counterstain. Eight non-overlapping fields were photographed in each group using a light microscope (Eclipse 80i Nikon, Tokyo, Japan) equipped with a digital camera (Diagnostic Instruments, Inc., Sterling Heights, MI) and analyzed with Image J software (National Institute of Mental Health, Bethesda, MD). At least three independent experiments were performed.

### Immunocytochemical analysis of autophagosomal-associated LC3 expression in rMC-1 cells

Assessment of autophagosomes in rMC-1 cells was performed by immunocytochemistry of LC3 expression. Briefly, cells were grown on a chamber slide and treated with CoCl_2_ (300μM) and NH_4_Cl (20mM) as well as Lutein (20μM) for 24 hours. The cells were then fixed with ice-cold methanol for 15 minutes at -20°C. After washes by PBS, cells were permeabilized with 0.3% Triton X-100 for 2 minutes. After blocking with 5% serum for 1 hour, the cells were incubated with LC3 antibody (1:400; Cell Signaling Technology, Beverly, MA) overnight at 4°C. After incubation with anti-Rabbit IgG FITC-conjugated secondary antibody, cell nuclei were counterstained by 4’,6-diamidino-2-phenlindole (DAPI). Eight nonoverlapping fields in each well were captured using a light microscope (Eclipse 80i Nikon, Tokyo, Japan) equipped with a digital camera (Diagnostic Instruments, Inc., Sterling Heights, MI) and analyze with Image J software (National Institute of Mental Health, Bethesda, MD). At least three independent experiments were performed.

### Statistical Analysis

Data were presented as mean ± SEM and analyzed using Prism v4.0. (GraphPad software Inc., San Diego, CA). ANOVA followed by Bonferroni multiple comparison tests was used for all data analysis. P value less than 0.05 was set as statistically significant.

## Results

### Lutein ameliorated cobalt (II) chloride (CoCl_2_)-induced hypoxic cell death in cultured rat Műller cells (rMC-1)

**Fig 1:**
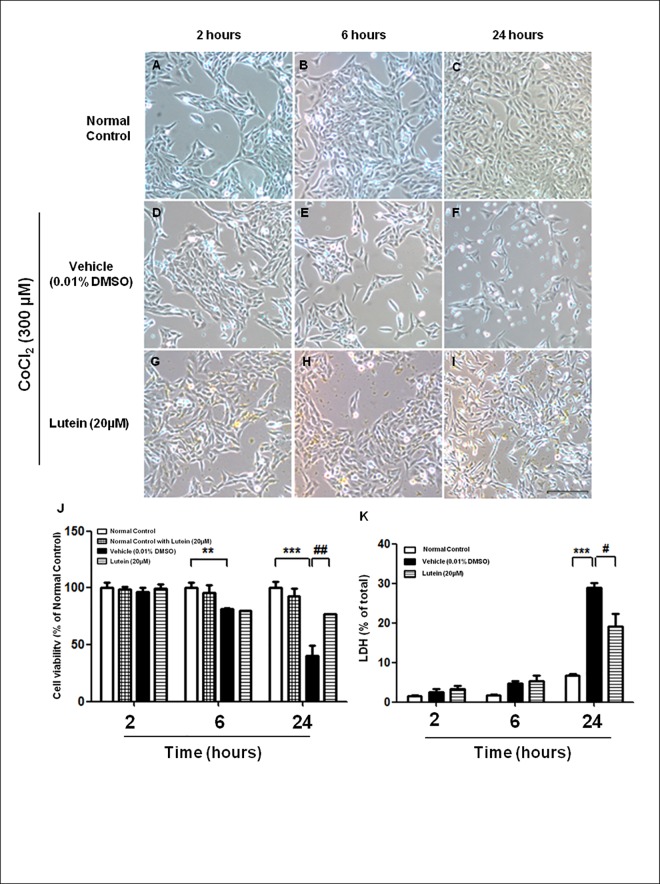
Lutein rescued rMC-1 cells from CoCl_2_-induced hypoxic injury. rMC-1 cells were exposed to CoCl_2_ (300μM) with or without lutein for various periods. Representative photographs of rMC-1 cells **(A-C)** normal control, **(D-F)** hypoxia with vehicle (0.01% DMSO), **(G-I)** hypoxia with lutein (20μM). **(J)** Percentage of cell viability. Treatment of lutein only without hypoxia did not affect the viability when compared with the normal control. Lutein-treated rMC-1 cells showed higher cell viability when compared with the vehicle-treated group at 24 hours. **(K)** Percentage of lactate dehydrogenase (LDH) release from damaged cells. Lutein attenuated LDH release after CoCl_2_-induced injury when compared with that in vehicle-treated group at 24 hours. n = 5 in each group. ^**^P< 0.01, ^***^P<0.001 versus normal control group; ^#^*P*< 0.05, ^##^*P*<0.01 versus vehicle-treated group. Scale bar, 100 μm.

**Fig 2:**
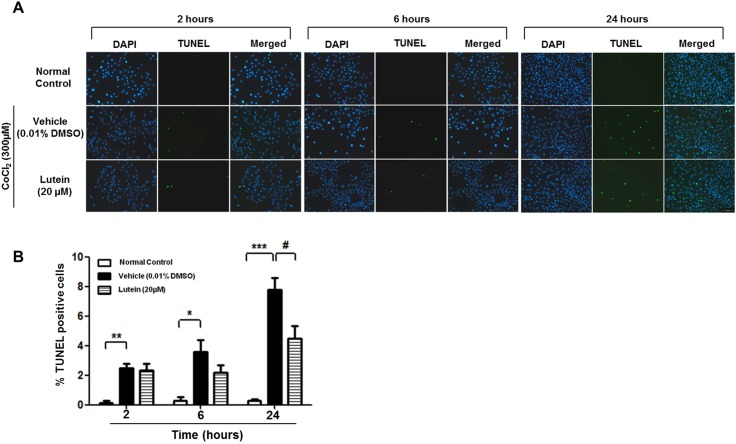
Number of apoptotic nuclei was attenuated in lutein-treated cells. Apoptotic nuclei were revealed by TUNEL assay after CoCl_2_ treatment at different time points. **(A)** Representative images of TUNEL-positive nuclei (green) and DAPI-stained nuclei (blue) in rMC-1 cells. **(B)** Quantification of TUNEL-positive cells. n = 5 in each group. Lutein administration significantly reduced the number of TUNEL-stained nuclei. Scale bar, 100 μm. ^*^P<0.05, ^**^P< 0.01, ^***^P<0.001 versus normal control group; ^#^*P*< 0.05 versus vehicle-treated group.

To mimic the hypoxic/ischemic condition, 300μM of CoCl_2_ was used to induce chemical hypoxia in rMC-1 cells [19,21]. The morphology of vehicle-treated rMC-1 cells started to change 6 hours after the CoCl_2_ treatment when compared with that in the normal control (Fig 1E and 1F). Cells became round in shape and cytoplasmic vacuoles were observed (Fig 1E and 1F). On the other hand, the morphology of lutein-treated cells remained similar to that in the normal control despite the exposure to CoCl_2_ for 24 hours (Fig 1I). rMC-1 cells treated with lutein alone (20 μM) did not show any significant difference in cell viability when compared with normal control in all time periods tested. On the other hand, viability of vehicle-treated rMC-1 cells started to decline at 6 hours after CoCl_2_ treatment; there was approximately 50% loss of cell viability after hypoxic challenge for 24 hours (Fig 1J). Lutein-treated rMC-1 cells were also affected upon CoCl_2_-induced injury, resulting in around 20% reduction of cell viability. However, viability of lutein-treated rMC-1 cells were not further affected upon prolonged CoCl_2_ treatment (Fig 1J). CoCl_2_-induced cytotoxicity in rMC-1 cells also led to release of lactate dehydrogenase (LDH) at 24 hours after hypoxic challenge; yet, LDH release was attenuated in lutein-treated rMC-1 cells (Fig 1K). To further assess if CoCl_2_-induced cytotoxicity was associated with apoptosis, TUNEL staining was performed to identify the apoptotic nuclei in rMC-1 cells (Fig 2A) and their numbers were counted. Percentage of apoptotic nuclei started to elevate at 2 hours after incubation with CoCl_2_ and continued to rise until 24 hours in vehicle-treated rMC-1 cells, indicating that CoCl_2_ induced apoptosis-related cell death in a time-dependent manner (Fig 2B). The percentage of apoptotic nuclei was decreased after lutein treatment for 24 hours when compared with the vehicle-treated rMC-1 cells. This finding is consistent with the cell viability assay that lutein rescued rMC-1 cells from CoCl_2_-induced hypoxia at 24 hours.

### Lutein protected rMC-1 cells from hypoxic-induced cell death by altering expression of apoptosis-associated proteins

**Fig 3:**
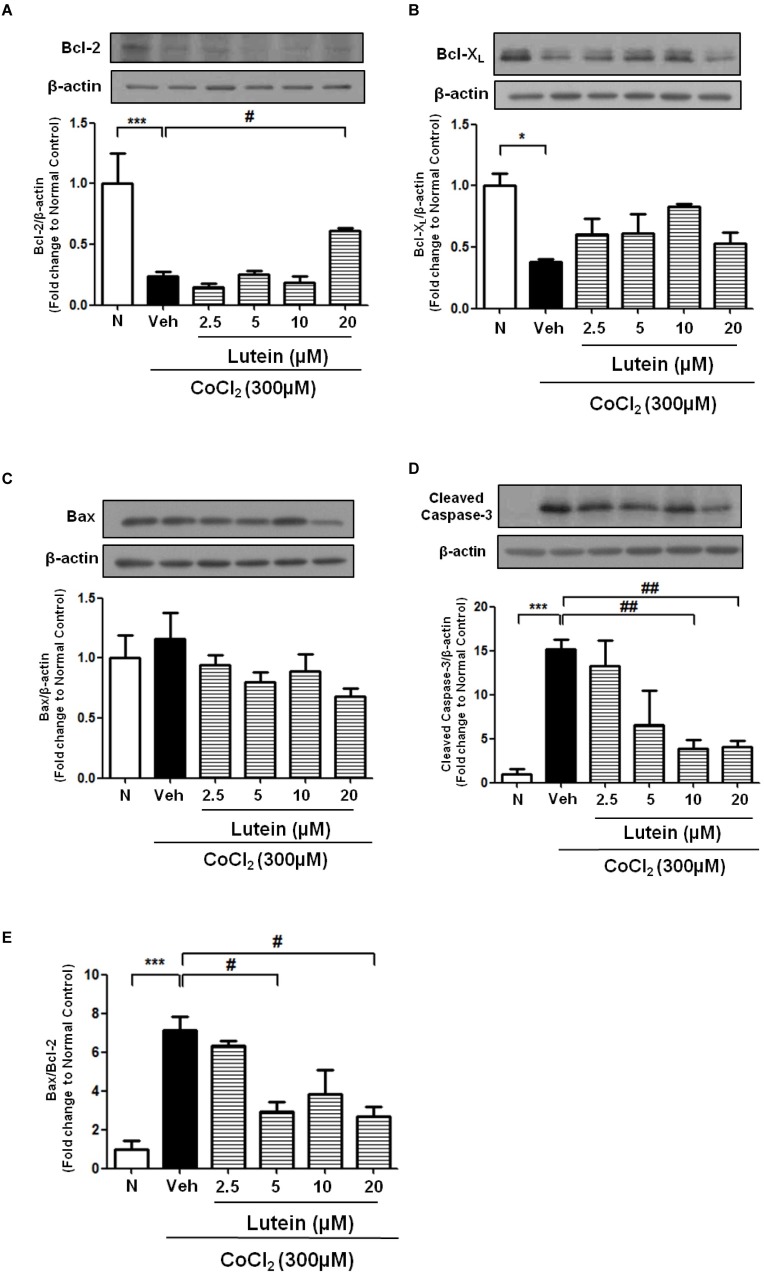
Anti-apoptotic effects of lutein in rMC-1 cells upon CoCl_2_-induced hypoxia. rMC-1 cells were exposed to CoCl_2_ (300μM) with various concentration of lutein for 24 hours. **(A-D)** Protein levels of different apoptotic-related proteins including Bcl-2, Bcl-X_L_, Bax, and cleaved caspase 3 were measured by Western blotting (normalized by β-actin) and quantified by densitometry. 20μM of Lutein was able to up-regulate protein expression of Bcl-2 and protein level of cleaved caspase 3 was inhibited by both 10μM and 20μM of lutein. **(E)** Densitometry analysis of ratio of Bax and Bcl-2 protein expression with different concentration of lutein. Lutein improved rMC-1 cell survival by decreasing Bax/Bcl-2 ratio. n = 5 in each group. ^*^P<0.05, ^***^P<0.001 versus normal control group; ^#^*P*< 0.05, ^##^*P*<0.01 versus vehicle-treated group. N, Normal Control; Veh, Vehicle (0.01% DMSO).

**Fig 4:**
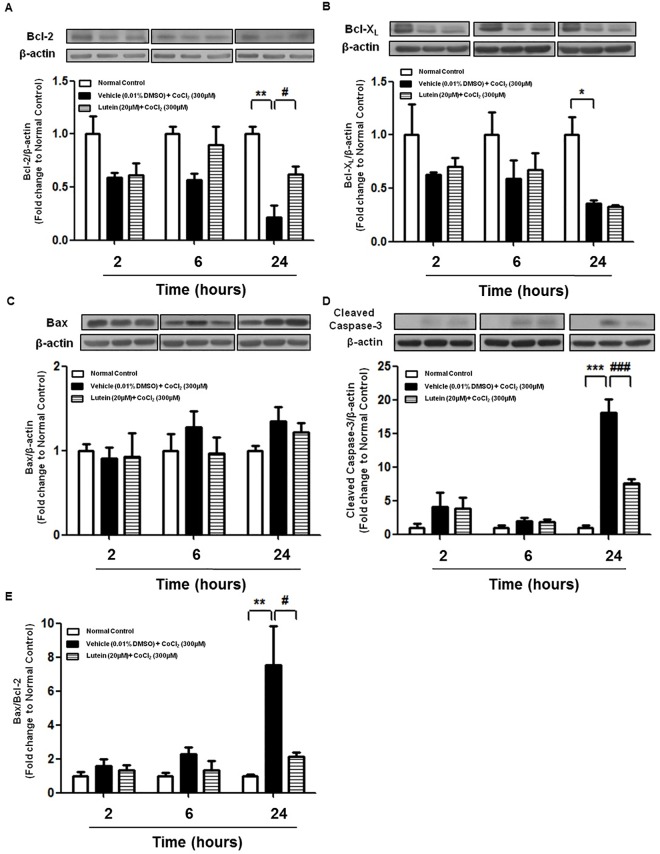
The length of lutein treatment in rMC-1 cells upon CoCl_2_-induced hypoxia. rMC-1 cells were exposed to CoCl_2_ (300μM) with or without lutein (20μM) for different periods of time. **(A-D)** Protein levels of different apoptotic-related proteins including Bcl-2, Bcl-X_L_, Bax, and cleaved caspase 3 were measured by Western blotting (normalized by β-actin) and quantified by densitometry. Lutein treatment was able to rescue cells upon CoCl_2_-induced cell death by up-regulating protein expression of Bcl-2 and suppressing cleaved caspase 3 at 24 hours. **(E)** Densitometry analysis of ratio of Bax and Bcl-2 protein expression at different time points. Lutein improved rMC-1 cell survival by decreasing Bax/Bcl-2 ratio at 24 hours. n = 5 in each group. ^*^P<0.05, ^**^P< 0.01, ^***^P<0.001 versus normal control group; ^#^*P*< 0.05, ^###^*P*<0.001 versus vehicle-treated group.

The role of Bcl-2 family proteins in CoCl_2_-induced apoptosis and lutein-mediated protection was studied. Exposure of rMC-1 cells to CoCl_2_ significantly down-regulated the expression of two anti-apoptotic proteins, Bcl-2 and Bcl-X_L_ when compared with normal control, resulting in more than 50% reduction in their protein levels at 24 hours (Fig 3A and 3B; Fig 4A and 4B). However, expression of a pro-apoptotic protein, Bax, was shown a trend of increase upon CoCl_2_ treatment (Figs 3C and 4C). The ratio of Bax to Bcl-2 protein expression and cleaved caspase 3 were significantly up-regulated after incubation with CoCl_2_ for 24 hours in vehicle-treated cells (Fig 3D and 3E; Fig 4D and 4E). Protein level of Bcl-2 but not Bcl-X_L_ was enhanced by 20 μM of lutein at 24 hours while that of Bax remained unaffected (Fig 3A, 3B and 3C; Fig 4A, 4B and 4C). Furthermore, Bax/Bcl-2 ratio as well as cleaved caspase 3 was remarkably attenuated by 20μM of lutein upon CoCl_2_-induced hypoxia for 24 hours (Fig 3D and 3E; Fig 4D and 4E). Together, these data suggested that the Bcl-2 protein family was involved in lutein-mediated protection against CoCl_2_-induced apoptosis in rMC-1 cells.

### Lutein was involved in anti-autophagic mechanism in CoCl_2_ -induced autophagy in rMC-1 cells

**Fig 5:**
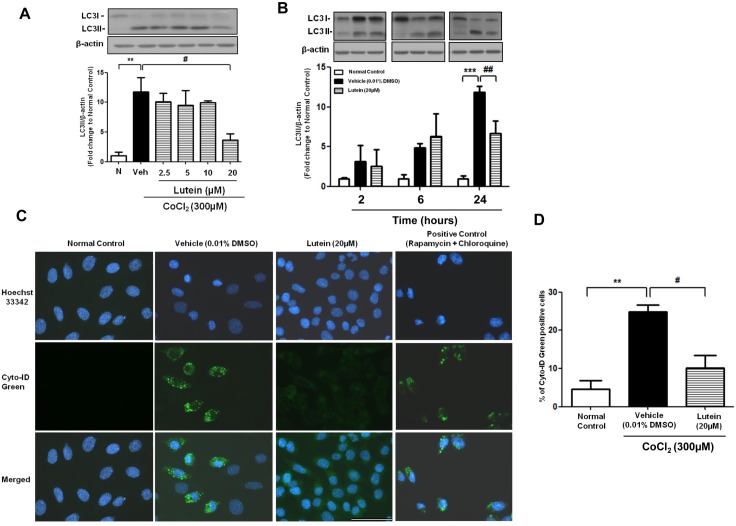
Lutein protected rMC-1 cells from CoCl_2_-induced autophagy. Western blot analysis of expression of an autophagic marker, LC3II, along with the densitometric quantification (normalized by β-actin). **(A)** rMC-1 cells were exposed to CoCl_2_ (300μM) together with various concentration of lutein for 24 hours. LC3II expression was up-regulated upon CoCl_2_-induced hypoxia and attenuated by 20μM of lutein. **(B)** Expression of LC3II was up-regulated 2 hours after CoCl_2_ treatment and continued to increase in the remaining time points. Lutein was able to attenuate LC3II expression at 24 hours after hypoxic challenge. **(C)** Autophagosome formation was monitored in rMC-1 cells 24 hours after CoCl_2_ treatment. Both normal control and treated cells were stained with Cyto-ID^®^ green dye and Hoechst 33342. Representative fluorescence microscopy images exhibited a decreased number of green fluorescence-labeled autophagosomes in lutein-treated cells when compared with that in vehicle-treated cells. Rapamycin and chloroquine co-treatment in rMC-1 cells were used as the positive control to identify the presence of autophagosome in the cells. **(D)** Quantification of Cyto-ID^®^ green positive cells. Data was presented as the percentage of Cyto-ID^®^ green positive cells over the total number of cells counted. n = 4 in each group. ^**^P< 0.01, ^***^P<0.001 versus normal control group; ^#^*P*<0.05, ^##^*P*<0.01 versus vehicle-treated group. Scale bar, 50 μm.

It has been reported that hypoxia- inducible factor 1 α (HIF-1α) was activated by CoCl_2_ and triggered hypoxic cell death and autophagy [22,24]. We therefore further investigated whether CoCl_2_ induced autophagy and caused the loss of viability in rMC-1 cells. rMC-1 cells were treated with 3-Methyladenine (3-MA), a selective autophagic inhibitor through suppressing the activity of class III PI3K (an autophagic initiator) and inhibiting the early stage of autophagosomal formation [25] before CoCl_2_ treatment for 24 hours. Viability of 3-MA pre-treated cells was higher when compared with the vehicle control upon hypoxic injury while 3-MA treatment only without hypoxia did not cause any significant difference in cell viability, indicating CoCl_2_-induced autophagy contributed to hypoxic cell death in rMC-1 cells (S1 Fig). To further monitor activation of autophagy, immunofluorescence analysis of the autophagosomes and immunoblotting of microtubule-associated protein light chain 3 (LC3) were performed [26]. LC3 comprises of two forms, the cytosolic LC3I and the lipidated LC3II. During onset of autophagy, cytosolic LC3I is modified into the LC3II and localized on the double-membrane of autophagosome. Thus, levels of LC3II are closely associated with abundance of autophagosome [27]. In our experiments, protein expression of LC3II was determined after CoCl_2_ treatment; it was activated at 2 hours after hypoxic injury and increased gradually until 24 hours (Fig 5B). Yet, this was reversed by the application of 20μM lutein at 24 hours (Fig 5A and 5B), indicating that lutein administration was able to attenuate the autophagic event upon CoCl_2_ treatment. Next, we examined autophagosome formation after 24 hours of CoCl_2_ exposure. Immunofluorescence analysis revealed that green punctate vacuoles were observed at the perinuclear region of the cells, indicating the presence of CoCl_2_-induced autophagy in rMC-1 cells (Fig 5C). This observation is consistent with the green-fluorescence labeled vacuoles as shown in the positive control cells, which were co-treated with rapamycin and chloroquine to induce autophagy (Fig 5C positive control). Lutein-treated rMC-1 cells, however, exhibited a lower percentage of green-labeled vacuoles comparable to those observed in the normal control (Fig 5D). The decreased number of autophagsomal related vacuoles is in agreement with the attenuated LC3II expression upon lutein administration. Accordingly, these data illustrated that CoCl_2_ induced autophagy as demonstrated by increasing LC3II expression and autophagosome formation and these can be reversed by 20μM of lutein for 24 hours.

### Lutein attenuated autophagosomes formation through regulating mTOR-mediated pathway

**Fig 6:**
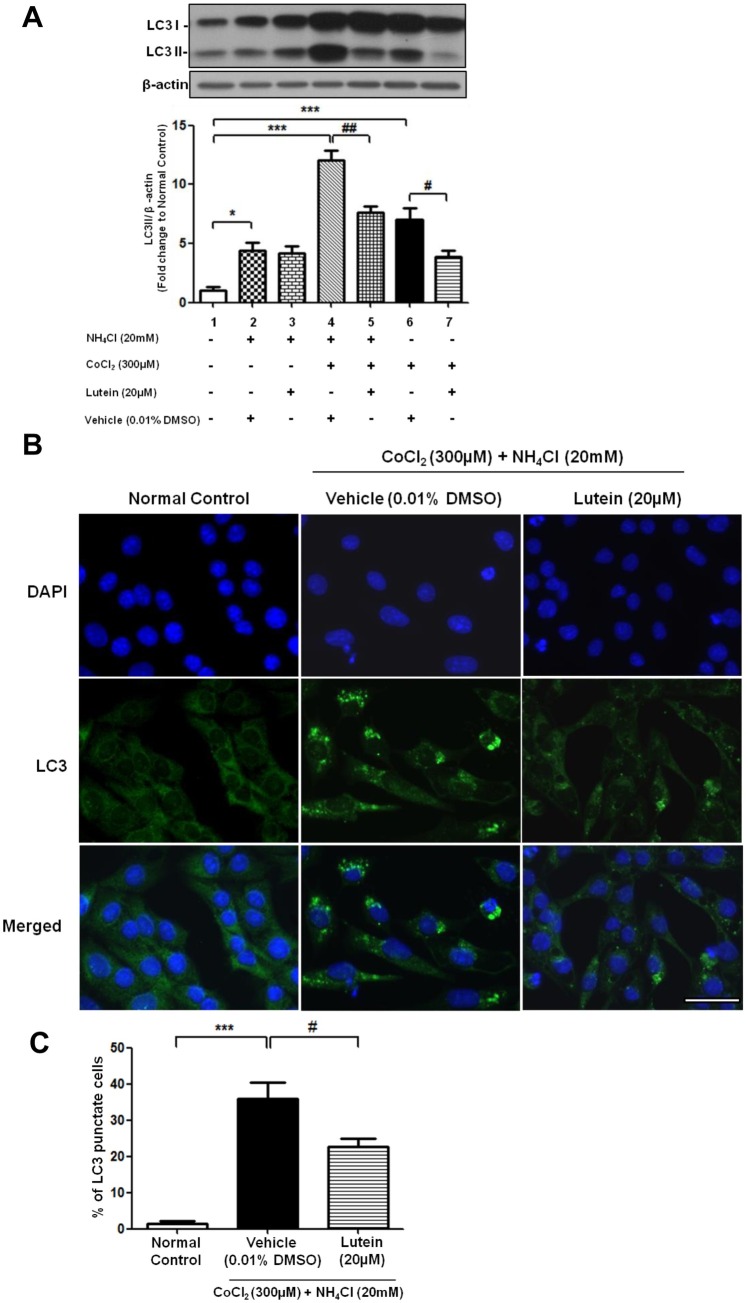
Lutein protected rMC-1 cells from CoCl_2_ –induced autophagy through reduction of autophagic flux. rMC-1 cells were treated with CoCl_2_ in the presence or absence of an autophagic flux inhibitor, ammonium chloride (NH_4_Cl), for 24 hours. **(A)** Western blotting and the densitometric quantification revealed that LC3II expression was activated after CoCl_2_ treatment in the presence of NH_4_Cl (lane 4) while lutein was able to attenuate this LC3II accumulation (lane 5). **(B)** Representative fluorescence microscopy images showed an increased number of LC3 punctate cells in CoCl_2_ and NH_4_Cl co-treated cells. Administration of lutein alleviated the puncate cells upon CoCl_2_ treatment. **(C)** Quantification of number of LC3 puncate cells. Data was presented as the percentage of LC3 puncate cells over the total number of cells counted. n = 5 in each group. ^*^P< 0.05, ^***^P<0.001 versus normal control group; ^##^*P*< 0.01 versus NH_4_Cl and CoCl_2_ co-treatment group; ^#^*P*< 0.05, versus vehicle-treated group. Scale bar, 50 μm.

Up-regulation of the autophagic marker by hypoxic injury can be due to either the increase of autophagic flux or reduction of autolysosome degradation. Treatment with lysosomotropic agent ammonium chloride (NH_4_Cl) in rMC-1 cells resulted in an increased protein expression of LC3II, suggesting the blockade of autophagosome-lysosome fusion led to accumulation of autophagosomes (Fig 6A, lane 2). When cells were co-treated with CoCl_2_ and NH_4_Cl for 24 hours, LC3II expression was further elevated when compared with cells treated with CoCl_2_ alone (Fig 6A, lane 4). These data depicted that increased level of LC3II by CoCl_2_ is due to the enhancement of autophagic flux rather than reduced clearance of autophagosome. Furthermore, co-treatment of CoCl_2_ and NH_4_Cl for 24 hours showed an accumulation of punctate form of LC3 in rMC-1 cells (Fig 6B). To further investigate whether lutein is involved in attenuation the flux of autophagosome formation, rMC-1 cells were treated with lutein together with NH_4_Cl and CoCl_2_ for 24 hours. LC3II expression as well as the percentage of LC3 punctate cells was significantly decreased when compared with those without lutein treatment, suggesting that lutein reduced autophagosomal flux in CoCl_2_-induced autophagy in rMC-1 cells (Fig 6A lane 5 and 6C).

**Fig 7:**
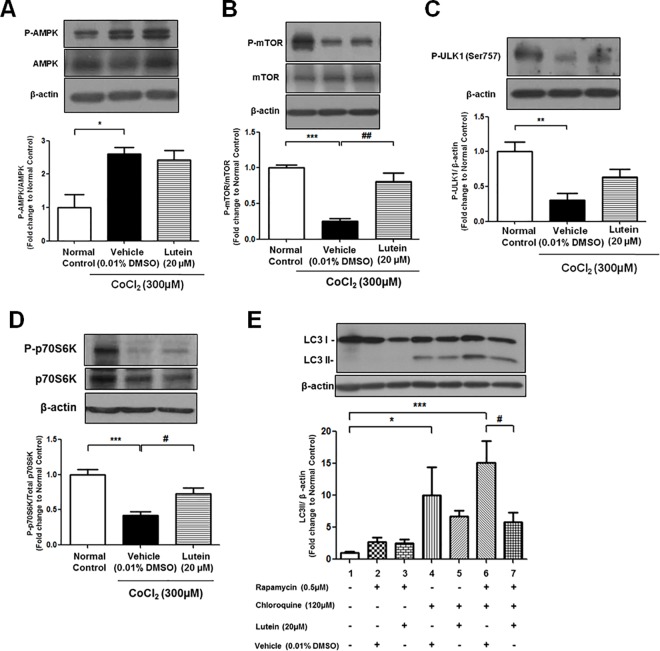
Anti-autophagic property of lutein was involved in mTOR-mediated autophagy pathway. **(A)** Western blotting and the densitometric analysis showed that the phosphorylated AMPK was up-regulated in CoCl_2_-treated cells. **(B-D)** Protein levels of mTOR-associated proteins including P-mTOR, P-p70S6K and P-ULK1 (Ser757) were measured by Western blotting (normalized by β-actin) and quantified by densitometry. 20μM of lutein was able to restore the phosphorylation levels of P-mTOR and P-p70S6K upon CoCl_2_-induced hypoxia. (E) Rapamycin was used to induce autophagy and chloroquine was also added to block the formation of autolysosome upon rapamycin-mediated autophagy. Densitometry analysis showed that LC3II protein expression was up-regulated in rapamycin-induced autophagy and accumulated in the presence of chloroquine (lane 6). Lutein treatment was able to decrease LC3II expression in rMC-1 cells upon rapamycin and chloroquine co-treatment (lane 7). n = 5 in each group. ^*^P< 0.05, ^**^P<0.*01*, ^***^P<0.001 versus normal control group; ^#^*P*< 0.05, ^##^*P*<0.01 versus Lutein-treated group; ^#^*P*< 0.05, rapamycin and chloroquine co-treatment group.

Previous study reported that treatment of CoCl_2_ triggered an inhibition of mTOR pathway, leading to autophagy in cardiomyoblasts [22]. To examine the involvement of mTOR-asscoiated pathway upon CoCl_2_-induced hypoxia, protein expression of phosphorylated AMP-activated protein kinase (AMPK) which is a key energy sensor and functions as a negative regulator to mTOR was detected [28]. Phosphorylation of AMPK was remarkably up-regulated upon CoCl_2_ treatment for 24 hours, suggesting that AMPK phosphorylation was triggered upon hypoxia in rMC-1 cells (Fig 7A). Furthermore, activation of AMPK caused by CoCl_2_ treatment for 24 hours led to suppression of the phosphorylation of mTOR, p70S6K and ULK1 (Ser757), strengthening the notion that inhibition of mTOR pathway was induced upon CoCl_2_ treatment (Fig 7B, 7C and 7D). However, phosphorylation activities of both mTOR and p70S6K were restored in lutein-treated cells (Fig 7B and 7D), depicting that mTOR pathway is a possible pathway in lutein-mediated anti-autophagic effect. To further validate this finding, rMC-1 cells were treated with rapamycin, which has been used to inhibit mTOR and induce autophagy [26]. Activation of rapamycin-induced autophagy was determined by protein expression of LC3II. As shown in Fig 7E, incubation with rapamycin slightly up-regulated LC3II expression. To prevent lysosomal degradation of LC3II in rapamycin-treated cells, cells were treated with chloroquine to impair the LC3II protein turnover in the autolysosome. Accumulation of autophagosomes was found in cells concomitantly treated with rapamycin and chloroquine, as measured by the autophagic vacuoles formation by immunofluorescence analysis and LC3II expression by Western blotting (Fig 5C positive control and Fig 7E, lane 6). When rMC-1 cells were exposed to both rapamycin and chloroquine in the presence of lutein, LC3II expression was remarkably attenuated to nearly the basal level of chloroquine-induced LC3II (Fig 7E, lane 7). Together, these results suggest that lutein attenuates autophagic flux from the mTOR-mediated autophagy in rMC-1 cells.

## Discussion

Retinal ischemia/reperfusion (I/R) injury has been associated with different ocular diseases such as diabetic retinopathy leading to irreversible neuronal death. Lutein belongs to the family of xanthophylls and is known as a potent anti-oxidant. It protects the macula from the damage of high energy blue light [12]. Our previous studies demonstrated the neuroprotective effects of lutein in the retinal ischemic animal model [1,21,29]. We found that lutein protected retinal neuron and attenuated Műller cell gliosis from I/R injury by its anti-oxidative and anti-inflammatory properties [1,21]. Moreover, lutein could improve the viability of retinal neurons from CoCl_2_-induced hypoxia *in vitro* [19] while the protective effects of lutein were also observed in other *in vitro* models [30–32]. In the present study, lutein increased cell viability and decreased LDH release after CoCl_2_ treatment. Lutein also attenuated the number of TUNEL positive cells upon hypoxia, in agreement with our previous study that lutein reduced apoptotic nuclei after retinal I/R injury [1].

CoCl_2_ have been commonly used *in vitro* to mimic the hypoxic/ischemic condition. It induces reactive oxygen species (ROS) generation and leads to cell death. It has been reported that ROS was induced by retinal ischemia and resulted in retinal cell death [17]. Although the CoCl_2_ model only partly mimics the hypoxic condition, our earlier study clearly showed the protective effects of lutein in Műller cells *in vitro* [21]. Another hypoxic model that is also commonly used is the oxygen-glucose deprivation (OGD), achieved by culturing cells in glucose-free medium under low oxygen environment [20].

Several studies depicted that CoCl_2_ could alter protein expression of Bcl-2 family [33] and trigger caspase-cascade apoptosis [34]. In our study, CoCl_2_ treatment suppressed the expression of anti-apoptotic proteins Bcl-2 and Bcl-X_L_. Although the expression of pro-apoptotic protein Bax remained unaffected, there have been an increase of Bax/Bcl-2 ratio, an indicator of intrinsic apoptotic pathway [35], followed by activation of cleaved caspase 3, suggesting that CoCl_2_-induced hypoxia triggered this pathway. Our previous findings demonstrated that 20μM of lutein effectively suppressed the release of pro-inflammatory factors upon CoCl_2_-induced hypoxic challenge in rMC-1 cells [21]. In the present study, we showed that treatment of 20μM of lutein also enhanced Bcl-2 protein expression and abrogated Bax/Bcl-2 ratio after 24 hours of hypoxic injury. More importantly, lutein attenuated cleavage of caspase 3, consistent with our previous finding using an animal model of retinal detachment [36], further strengthening the notion that lutein mediated cytoprotection through regulating the cascade of caspase-associated pathway.

The beneficial or detrimental effects of autophagy in ischemic injury remain controversial. However, activation of autophagy in ganglion cell layer after retinal I/R injury has been documented differently, leading to ganglion cell death [4,37]. Most importantly, there is increasing evidence that inhibition of autophagy is protective in various *in vivo* I/R models [38–40]. Moreover, several *in vitro* studies also demonstrated that suppression of both apoptosis and autophagy enhanced cell survival [22,41]. Apart from apoptosis, CoCl_2_-induced hypoxia also triggered autophagy in rat cardiomyoblasts (H9c2) and retinal ganglion cells (RGC-5) cell line [22,42]. A study reported that the anti-apoptotic protein Bcl-2 acts as a pro-survival protein and negatively regulates autophagy, indicating the crosstalk between apoptosis and autophagy [43]. Here, we also showed that CoCl_2_ induce not only apoptosis but also autophagy in rMC-1 cells. Inhibition of autophagy using a pharmacological approach with 3-MA prevented the loss of viability against CoCl_2_-induced hypoxic injury, suggesting that the autophagic event contributed to CoCl_2_-induced cell death. Our findings also revealed that protein level of Bcl-2 was suppressed by CoCl_2_ treatment but its expression was enhanced in lutein-treated cells, suggesting that restoration of the pro-survival protein Bcl-2 might partly contribute to the protective effects of lutein against both apoptosis and autophagy. In addition, expression of lipidated form LC3II and autophagic vacuoles was attenuated in lutein-treated rMC-1 cells, suggesting that lutein might be involved in alleviation of autophagy provided that LC3II abundance is associated with formation of autophagosomes [27]. Moreover, accumulation of LC3 punctate cells were diminished in lutein-treated groups, further depicting our findings that lutein reduced autophagosome formation through reducing the autophagic flux.

It is known that mTOR is an important regulator for cell growth and metabolism; disruption of mTOR activity might contribute to cell death [44]. More importantly, mTOR negatively regulates autophagy through the phosphorylation of ULK1 (Ser 757), preventing the formation of ULK complex and subsequently inhibit autophagosome formation [28,45]. AMPK is an essential energy sensor that helps to maintain cellular homeostasis and it negatively regulates mTOR hence induces autophagy [28]. Our findings clearly showed that phosphorylated form of AMPK was up-regulated in CoCl_2_-treated rMC-1 cells while phosphorylation of mTOR and its substrate p70S6K was inhibited, suggesting that abrogation of mTOR activity is associated with CoCl_2_-induced autophagy. Phosphorylation of ULK1 (Ser 757) was also suppressed upon CoCl_2_ treatment, indicating that CoCl_2_ triggers autophagy by inhibition of mTOR pathway and de-phosphorylation of ULK1. Lutein administration restored the phosphorylation of mTOR and p70S6K, indicating that reactivation of mTOR pathway may be associated with lutein-mediated anti-autophagic protection. Rapamycin is commonly used as the inhibitor of mTOR pathway and it suppresses the inhibitory function of mTOR, leading to the formation of ULK complex and activates autophagy. Here, rapamycin treatment triggered LC3II expression in rMC-1 cells and the autophagosome-associated LC3II further accumulated after impairment of lysosomal degradation by chloroquine, indicated by the increased number of green fluorescence-labeled autophagic vesicles. We further found that lutein effectively attenuated the protein expression of LC3II in rapamycin and chloroquine co-treated cells, suggesting that lutein is involved in mTOR-associated autophagy.

We together with others have reported the anti-apoptotic, anti-oxidative and anti-inflammatory effects of lutein in various *in vivo* and *in vitro* models of retinal diseases [16,21,46–49]. Here, we further strengthened the anti-apoptotic effects of lutein and its possible mechanistic pathway on protecting retinal cells using a chemical induced hypoxic cell model. Lutein therefore may be beneficial in eye diseases where apoptotic cell death has been reported such as glaucoma, diabetic retinopathy and retinopathy of prematurity [50–52]. Most importantly, we discovered a novel protective effect of lutein in preventing autophagosome formation upon hypoxia-induced autophagy. Suppression on the flux of autophagosome formation can thus protect the retinal cells from hypoxic damage. In fact, modulation of autophagy become a therapeutics target for treatment of ocular diseases such as glaucoma, diabetic retinopathy and age-related macular degeneration (AMD) [4,53,54] however, the safety and side effects of the anti-autophagic drug has been concerned [55]. Developments of novel anti-autophagic drugs that are more specific to autophagic pathway and clinically safer are emerged. Several studies documented that mTOR can be the therapeutic target in various ischemic diseases [56–58]. A recent proteomics study reported that mTOR pathway was suppressed after retinal I/R injury, further delineating the protective role of mTOR [59]. It has been reported that increasing mTOR phosphorylation was neuroprotective against ischemic brain injury [58] and this prompt us to speculate that lutein not only protects the retinal Műller cells from hypoxia-induced apoptosis but also autophagy, possibly by targeting mTOR-associated pathway and improving cell survival against hypoxic injury.

Műller cells are the major glial cell type in the retina, it maintains homeostasis and protects retinal ganglion cells from neurotoxicity [60]. Our previous studies using retinal ischemic animal model demonstrated that lutein protected retinal neuronal cell death as well as Műller cell gliosis [1,21]. Together with our earlier studies [1,19,21], the present study prompt us to speculate that lutein protects ganglion cell loss possibly through attenuating apoptosis and autophagy in glial cell. Taken together, lutein has been shown to be safe as a daily supplement for ocular health with minimal side effects, its therapeutic potential as a clinically safe-to-be used protectant is strongly indicated.

## Supporting Information

S1 Fig Autophagy was involved in CoCl_2_-induced cell death in rMC-1 cells. Percentage of cell viability. 3-MA-pretreated rMC-1 cells showed higher viability when compared with the vehicle-treated group after CoCl_2_ for 24 hours. Treatment of 3-MA only without hypoxia did not affect the viability when compared with the normal control. n = 5 in each group. ^***^P<0.001 versus normal control group; ^#^*P*< 0.05 versus vehicle-treated group.
(TIF)
