# Supplementary figures and images for: Lutein Attenuates Both Apoptosis and Autophagy upon Cobalt (II) Chloride-Induced Hypoxia in Rat Műller Cells
Source: PLoS One. 2016 Dec 9;11(12):e0167828. doi: 10.1371/journal.pone.0167828 (PMC5148028; doi:10.1371/journal.pone.0167828)

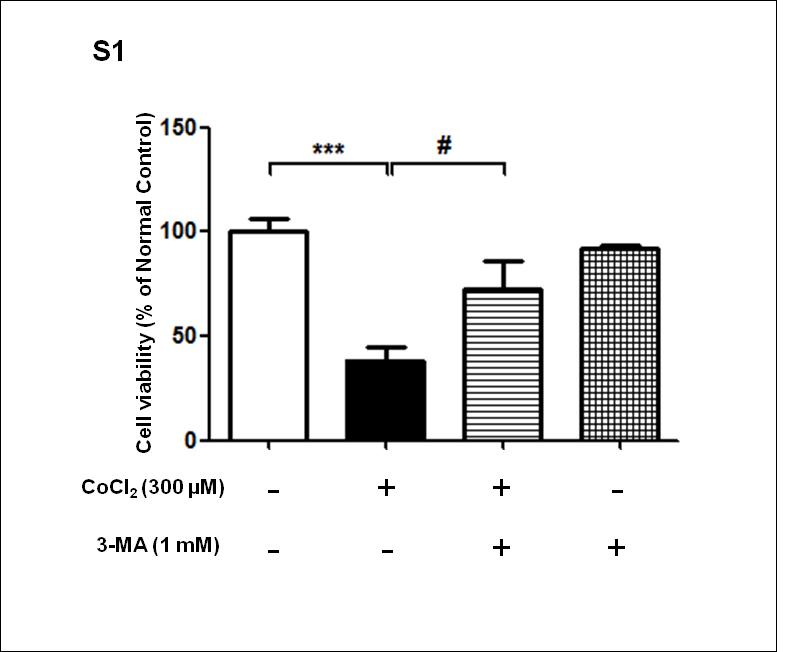

Supplement: S1 Fig — Percentage of cell viability. 3-MA-pretreated rMC-1 cells showed higher viability when compared with the vehicle-treated group after CoCl2 for 24 hours. Treatment of 3-MA only without hypoxia did not affect the viability when compared with the normal control. n = 5 in each group. ***P<0.001 versus normal control group; #P< 0.05 versus vehicle-treated group. (TIF) [file pone.0167828.s001.tif]
